# Cervical vagal nerve stimulation impairs glucose tolerance and suppresses insulin release in conscious rats

**DOI:** 10.14814/phy2.13953

**Published:** 2018-12-19

**Authors:** Harald M. Stauss, Hubert Stangl, Karen C. Clark, Anne E. Kwitek, Vitor A. Lira

**Affiliations:** ^1^ Department of Biomedical Sciences Burrell College of Osteopathic Medicine Las Cruces New Mexico; ^2^ Department of Health and Human Physiology The University of Iowa Iowa City Iowa USA; ^3^ Laboratory of Experimental Rheumatology University Hospital of Regensburg Regensburg Bayern Germany; ^4^ Department of Pharmacology The University of Iowa Iowa City Iowa USA

**Keywords:** Blood pressure, fasted blood glucose, glucagon, glucose tolerance test, heart rate, heart rate variability, neuromodulation

## Abstract

Previously, we reported that cervical vagal nerve stimulation (VNS) increases blood glucose levels and inhibits insulin secretion in anesthetized rats through afferent signaling. Since afferent signaling is also thought to mediate the therapeutic effects of VNS in patients with therapy‐refractory epilepsy and major depression, the question arises if patients treated with VNS develop impaired glucose tolerance. Thus, we hypothesized that cervical VNS impairs glucose tolerance in conscious rats. Rats (*n* = 7) were instrumented with telemetric blood pressure sensors and right‐ or left‐sided cervical vagal nerve stimulators (3 V, 5 Hz, 1 msec pulse duration, 1 h on 1 h off). Glucose tolerance tests (GTTs, 1.5 g dextrose/kg BW, i.p.) were performed after overnight fasting with the stimulators on or off (sham stimulation) in randomized order separated by 3–4 days. Overnight VNS did not alter mean levels of blood pressure or heart rate, but increased fasted blood glucose levels (140 ± 13 mg/dL vs. 109 ± 8 mg/dL,* P* < 0.05). The area under the blood glucose concentration curves of the GTTs was larger during VNS than sham stimulation (3499 ± 211 mg/dL*h vs. 1810 ± 234 mg/dL*h, *P* < 0.05). One hour into the GTTs, the serum insulin concentrations had decreased during VNS (−0.57 ± 0.25 ng/mL,* P* < 0.05) and increased during sham stimulation (+0.71 ± 0.15 ng/mL,* P* < 0.05) compared to the fasted baseline levels. These results demonstrate that chronic cervical VNS elevates fasted blood glucose levels and impairs glucose tolerance likely through inhibition of glucose‐induced insulin release in conscious rats. It remains to be determined if patients treated with VNS are at greater risk of developing glucose intolerance and type 2 diabetes.

## Introduction

Vagal nerve stimulation (VNS) has been considered a potential treatment option for patients with a variety of diseases including obesity (Masi et al. 2018), heart failure (De Ferrari et al. 2011; Premchand et al. 2014; Zannad et al. 2015; Gold et al. 2016), chronic pain (Chakravarthy et al. 2015), migraine (Grimsrud and Halker Singh 2018), and tinnitus (De Ridder et al. 2014). Based on the anti‐inflammatory effects of the parasympathetic nervous system (Tracey 2002; Komegae et al. 2018), currently explored therapeutic targets for VNS also include a variety of inflammatory diseases, such as rheumatoid arthritis (Koopman et al. 2017) and Crohn's disease (Bonaz et al. 2016). Currently, VNS is FDA‐approved for the treatment of therapy‐refractory epilepsy (Nune et al. 2015) and major depression (Cristancho et al. 2011). In these conditions the cervical vagus nerve is chronically stimulated through an implanted device (Giordano et al. 2017) and the therapeutic effects are thought to be mediated through afferent signaling to the central nervous system (Groves and Brown 2005; Krahl and Clark 2012). However, in a previous study (Meyers et al. 2016), we demonstrated that afferent signaling evoked by cervical VNS inhibits insulin secretion and markedly increases resting blood glucose levels in anesthetized rats, raising the question if patients treated with cervical VNS are at risk of developing glucose intolerance. Specifically, selective afferent cervical VNS (achieved by stimulating the cranial end of the sectioned cervical vagus nerve) caused a marked and sustained increase in blood glucose levels without concomitant increase in insulin serum concentration. The same hyperglycemic response was observed during stimulation of the intact cervical vagus nerve that was not dissected and, thus consisted of combined afferent and efferent VNS. In contrast, selective efferent stimulation (achieved by stimulating the peripheral end of the sectioned cervical vagus nerve) caused a small temporary increase in blood glucose concentration followed by an increase in serum insulin levels. The latter finding is consistent with a study by Peitl et al. (2005) that also demonstrated an increase in insulin plasma levels during electrical stimulation of the peripheral end of the cervical vagus nerve in anesthetized rats. Afferent signaling was not tested in this study because the cranial end or the intact vagus nerve was not stimulated. Thus, the conclusion of our previous study (Meyers et al. 2016) was that selective efferent VNS may potentially be effective in treating type 2 diabetes through stimulation of pancreatic insulin release, while stimulating the intact nerve (combined efferent and afferent VNS) may reduce glucose tolerance by suppression of insulin release via afferent VNS. Indeed, studies in animals suggest that chronic stimulation of vagal nerve branches other than the cervical vagus nerve may have beneficial effects on glucose metabolism. For example, chronic bilateral stimulation of the subdiaphragmatic vagal nerves improved insulin sensitivity in diet‐induced obesity in mini‐pigs (Malbert et al. 2017) and transcutaneous auricular vagus nerve stimulation prevented the increase in blood glucose levels and glycosylated HbA1c in Zucker diabetic fatty rats (Li et al. 2014). Thus, it is possible that stimulation of more peripheral branches of the vagus nerve (e.g., subdiaphragmatic) improves glucose metabolism through efferent signaling to metabolic end‐organs, such as the pancreas or the liver.

However, in the majority of the patients treated with VNS, stimulation occurs at the site of the cervical vagus nerve and both, efferent and afferent nerve fibers are activated. Thus, despite the reports from animal studies suggesting beneficial metabolic effects of VNS at more peripheral sites than the cervical vagus nerve (Li et al. 2014; Malbert et al. 2017) or with selective efferent cervical VNS (Peitl et al. 2005; Meyers et al. 2016), the question if non‐selective (combined efferent and afferent) VNS at the site of the cervical vagus nerve may deteriorate glucose tolerance and place patients at risk for type 2 diabetes is highly relevant and important because our previous study demonstrated inhibition of insulin release despite marked increases in blood glucose levels during non‐selective (intact nerve) and selective afferent (dissected nerve) cervical VNS. To further explore this question, we hypothesized that chronic cervical VNS of the intact vagus nerve inhibits glucose‐induced insulin release and, thus, impairs glucose tolerance in conscious rats. To test this hypothesis, we performed glucose tolerance tests during VNS and sham stimulation in conscious chronically instrumented rats. Sectioning the vagus nerve to achieve selective afferent or efferent VNS required us to perform our previous study (Meyers et al. 2016) during anesthesia. Anesthesia can potentially inhibit insulin release (Desborough et al. 1993). Thus, it was important to test the hypothesis of our current study in conscious animals.

## Methods

### Animals

A mixed population of aged (9.7 ± 0.7 months) male and female rats from various Lyon strains (Dupont et al. 1973) were used including congenic Lyon hypertensive rats with substituted fragments of chromosome 17 (Ma et al. 2017). These rat strains differ in cardiovascular and metabolic parameters (Vincent et al. 1993; Wang et al. 2015). Some of the rats were previously used as breeders whose offspring were used for another study (Ma et al. 2017). The characteristics of the individual animals included in this study are presented in Tables 1 and 2. The purpose of using a heterogeneous group of rats was not to investigate the impact of the various characteristics of the individual animals on the responses to VNS, but rather to test if the effect of cervical VNS on glucose metabolism is consistent among different strains of rats. Rats were housed in clear plastic cages, and temperature and light periods (12‐h light‐dark cycle; light in between 0600 h and 1800 h) were controlled. A standard rat chow (7912 or 2920X, Envigo, Madison, WI) and tap water were provided ad libitum, except during the nights before glucose tolerance tests, where food but not water was withheld. Experiments were approved by the Institutional Animal Care and Use Review Committee of the University of Iowa.

**Table 1:** Animal characteristics

Rat	Strain	Sex	Age (months)	BW (g)	VNS	BP_SYS_ (mmHg)	BP_Mean_ (mmHg)	BP_DIA_ (mmHg)	HR (bpm)
1	LN	F	11.8	242	Right	133	107	87	367
2	LL	F	9.0	280	Right	145	120	99	377
3	LL	F	8.7	274	Left	140	115	93	383
4	LH17LNConA2 het	F	10.1	320	Right	156	128	106	411
5	LH17LNConA3	F	10.6	296	Right	153	129	107	332
6	LH17LNConA3	M	6.6	572	Left	161	132	108	301
7	LH17LNConC2	F	11.1	364	Right	148	122	101	367

LN, Lyon normotensive rats; LL, Lyon hypotensive rats; LH17LNConXN, Lyon hypertensive rats, congenic for a snippet from LN rats on chromosome 17 (official strain names are provided in Table 2); F, female; M, male; BW, body weight at time of surgical instrumentation; VNS Right/Left, right or left cervical vagus nerve was stimulated; BP_SYS_, systolic blood pressure; BP_Mean_, mean blood pressure; BP_DIA_, diastolic blood pressure; HR, heart rate. Blood pressure and heart rate data are averages obtained with the stimulators turned off the night before the sham‐stimulated glucose tolerance tests.

**Table 2:** Official nomenclature of rat strains

Short name	Official nomenclature
LN	LN/MRrrcAek
LL	LL/MRrrcAek
LH17LNConA2 het	LH.LH‐Chr 17LN‐(rgdv421102132T‐rgdv413679765T)/Aek/LH/MRrrcAek (heterozygote)
LH17LNConA3	LH.LH‐Chr 17LN‐(Fanccrgdv551196202‐C‐rs107291522)/Aek
LH17LNConC2	LH.LH‐Chr 17LN‐(rs199194111‐rs105876746)/Aek

### Instrumentation

Rats were anesthetized using isoflurane (induction 5%, maintenance 1.5–2.5%). A telemetric blood pressure sensor (PA‐C40, Data Sciences International, St. Paul, MN) and a vagal nerve stimulator (model RNS, Harald Stauss Scientific, Iowa City, IA) were implanted as described previously (Stauss et al. 2014; Chapleau et al. 2016; Nizamutdinov et al. 2018). Briefly, the catheter of the telemetric blood pressure sensor was inserted in the right femoral artery and advanced into the abdominal aorta. The electrodes of the vagal nerve stimulator were wrapped around either the left or right cervical vagus nerve (randomly assigned to a side) and embedded in a silicone elastomer (Kwik‐Sil, World Precision Instrument, Inc., Sarasota, FL) for electrical insulation. Following surgical instrumentation, it was confirmed that the stimulators were in the “off” setting.

### Vagal nerve stimulation

**Figure 1:**
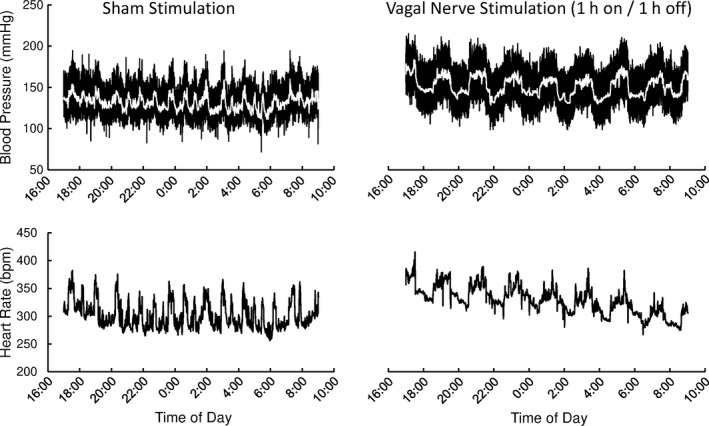
Telemetric recording of arterial blood pressure (top) and heart rate (bottom) during sham stimulation (left) and vagal nerve stimulation (VNS) (right) in an obese male hypertensive rat (# 6 in Table 1). Note the periodic fluctuations with a cycle length of 2 h with VNS caused by the stimulation protocol of cycles of 1 h of stimulation followed by 1 h without stimulation.

For VNS custom‐made stimulators (Model RNS, http://www.haraldstauss.com/HaraldStaussScientific) were used as described previously (Stauss et al. 2014; Chapleau et al. 2016; Nizamutdinov et al. 2018). The stimulators generated constant‐voltage, charge‐balanced rectangular impulses with a voltage of 3 V, a pulse duration of 1 msec, and a stimulation frequency of 5 Hz. These stimulation parameters are identical to the ones used in our previous study (Meyers et al. 2016). The mode of the stimulators could be switched from the “off” setting to a “continuous” stimulation mode and to a “cyclic” stimulation mode where the stimulators turned on and off every hour for a full cycle length of 2 h. During the night before glucose tolerance tests the stimulators were in the “cyclic” stimulation mode that allowed to verify proper function of the stimulators by the pronounced 2‐h oscillations in arterial blood pressure and heart rate induced by the “on” and “off” cycles as illustrated in Figure 1 (right). During the glucose tolerance tests the stimulators were switched to the “continuous” stimulation mode, to ensure continuous VNS throughout the duration of the glucose tolerance tests. For the sham‐stimulated condition, the stimulator was confirmed to be in the “off” setting the night before and during the glucose tolerance tests. Switching the stimulation mode of the implanted stimulators is possible using an external magnet. The stimulator setting was confirmed by a radio signal transmitted by the stimulators and received by an AM radio that indicates the current stimulator setting (“off”, “cyclic”, or “periodic”).

### Glucose tolerance tests

In each animal, two glucose tolerance tests (with sham stimulation and with VNS) were performed in the conscious condition, separated by three or four days. Thus, each rat was subjected to VNS and to sham stimulation, in random order. The first glucose tolerance test was performed three or four days following surgical instrumentation. In the afternoon (between 3:00 pm and 5:00 pm) on the day before a glucose tolerance test the vagal nerve stimulators were either turned on using the “cyclic” stimulation mode (stimulated condition) or left in the off position (sham‐stimulated condition). The order of the experimental conditions (stimulated or sham‐stimulated) was assigned randomly. At the same time, telemetric recording of arterial blood pressure and heart rate was started and continued till the end of the glucose tolerance test the next morning. Food was removed over night, but drinking water was provided. The next morning (between 8:30 am and 9:30 am) the stimulators were either switched to the “continuous” stimulation mode for the stimulated condition or the “off” setting was confirmed for the sham‐stimulated condition before the glucose tolerance tests were started. First, a blood sample was taken by puncturing the saphenous vein (conscious animals) for determination of baseline fasted blood glucose, serum insulin, and serum glucagon concentrations. Then 1.5 g dextrose/kg BW was injected intraperitoneally, followed by additional blood sampling at 15 min, 30 min, 60 min, 90 min, and 120 min after dextrose administration. Blood glucose levels were determined for all blood samples and serum insulin and glucagon concentrations were determined at the 60 min time point. Blood glucose levels were assessed using the TRUEtrack glucose meter (Nipro Diagnostics, Fort Lauderdale, FL) and serum insulin and glucagon concentrations were determined using commercial ELISA kits (Kit #90010 for insulin and Kit #81505 for glucagon, CrystalChem, Downers Gove, IL) as described previously (Meyers et al. 2016). Following the glucose tolerance tests, the vagal nerve stimulators were turned off or the “off” setting was confirmed. In one animal (Rat 1 in Table 1), we were unable to obtain adequate large volumes of blood for determination of insulin and glucagon. Thus, data presented in Table 4 and Figure 3 are based on six instead of seven animals.

### Heart rate variability analysis

From the telemetric arterial blood pressure recordings (duration 15–17 h, sampling rate 500 Hz), systolic, mean, and diastolic blood pressure and heart rate values were extracted and average values calculated for the entire duration of the recordings excluding the time period of the glucose tolerance tests. The derived heart rate time series were resampled at an equidistant sampling rate of 10 Hz and power spectra were calculated (segments of 4096 values with 50% overlap). The spectral powers were then calculated for very low frequency (VLF, 0.02–0.2 Hz), low frequency (LF, 0.2–0.8 Hz), and high frequency (HF, 1.0–3.0 Hz) ranges and for the total spectrum (0–5 Hz). Relative spectral powers were calculated as absolute spectral power divided by the difference of total power minus VLF power and expressed as percent. VNS occurred at cycles of 1 h stimulation followed by 1 h without stimulation (cycle length 2 h). To verify proper function of the stimulators the spectral power of the heart rate oscillations at a cycle length of 2 h was also assessed by computing the spectra from the 10 Hz heart rate time series without segmenting (1,048,576 values with zero padding as appropriate) and calculating the spectral power in the frequency range of 0.00010–0.00018 Hz, corresponding to a 2 h cycle. All computations were performed using the freely available HemoLab software (http://www.haraldstauss.com/HaraldStaussScientific/default.html).

### Statistics

Data are presented as means ± SEM. Glucose, insulin, and glucagon data obtained during glucose tolerance tests were analyzed by 2‐way analysis of variance (ANOVA) with experimental conditions (sham stimulation vs. VNS) and time points during the glucose tolerance tests as the two repeated factors (each animal underwent both, VNS and sham‐stimulation). In case of significance in the 2‐way ANOVA, post hoc tests were performed for comparison of time points (Scheffé test) and experimental conditions at each time point (paired t‐test with adjustment for multiple comparisons). For all other data, the Wilcoxon signed‐rank test for paired observations was used to compare the two experimental conditions. Statistical significance was assumed for *P* < 0.05.

## Results

### Hemodynamic and cardiac autonomic responses to VNS

Biological responsiveness to VNS was verified by pronounced blood pressure and heart rate oscillations with a cycle length of 2 h induced by the periodic stimulation protocol (Fig. 1, right) resulting in a pronounced peak in the heart rate spectrum at a frequency of 0.000139 Hz (2 h period) during VNS, but not during sham stimulation (Table 3). Compared to sham stimulation, VNS did not affect mean levels of heart rate (339 ± 12 vs. 362 ± 14 bpm), systolic (150 ± 8 vs. 148 ± 4 mmHg), mean (122 ± 7 vs. 122 ± 3 mmHg), or diastolic (99 ± 7 vs. 100 ± 3 mmHg) blood pressure. However, VNS increased low‐ and high‐frequency heart rate variability in absolute (*P* < 0.05) and relative (*P* < 0.10) units (Table 3) suggesting increased autonomic modulation of cardiac function.

**Table 3:** Heart rate spectral analysis

	LF absolute (bpm^2^)	LF relative (%)	HF absolute (bpm^2^)	HF relative (%)	Total power (bpm^2^)	2 h peak (bpm^2^)
Sham	7.2 ± 2.1	9.2 ± 2.5	0.54 ± 0.10	0.73 ± 0.13	124 ± 36	30 ± 13
VNS	31.7 ± 18.3*	13.1 ± 1.5^(^ * ^)^	5.13 ± 3.69*	1.61 ± 0.25^(^ * ^)^	330 ± 201	1,178 ± 688*

Absolute and relative low frequency (LF, 0.2–0.8 Hz) and high frequency (HF, 1.0–3.0 Hz) spectral power and total power of heart rate variability. In addition, the spectral power at a frequency corresponding to a 2‐h cycle (0.000139 Hz) is provided (2 h Peak). *P < 0.05 VNS versus Sham; ^(*)^ *P* < 0.10 VNS versus Sham.

### Effects of VNS on glucose tolerance

**Figure 2:**
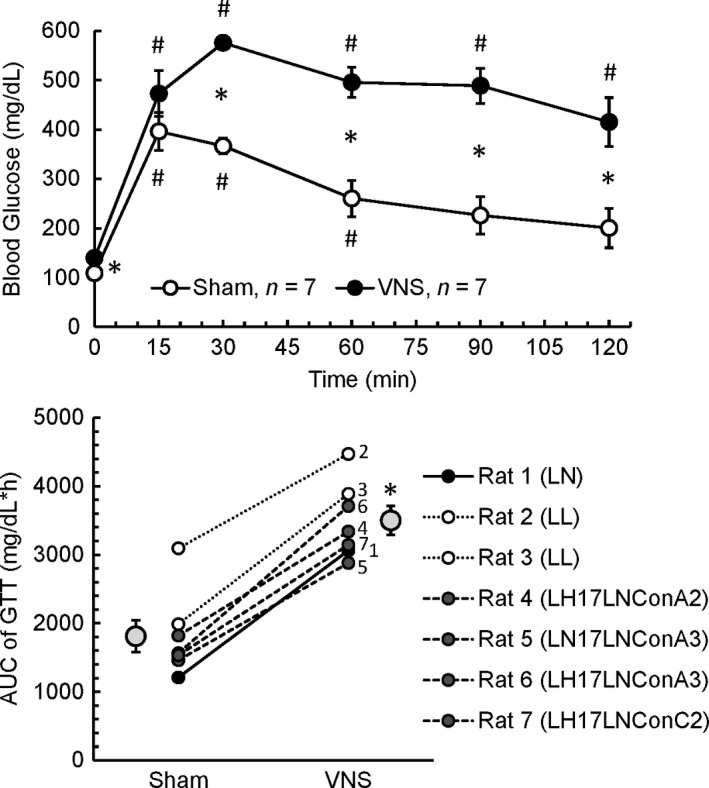
*Top:* Time course of blood glucose concentration during the glucose tolerance tests with sham stimulation (open circles) or vagal nerve stimulation (VNS, black circles). During VNS, the blood glucose concentration increased more than during sham stimulation. In contrast to sham stimulation, during VNS blood glucose concentration remained elevated compared to baseline values throughout the 120 min time period of the glucose tolerance tests. Values are means ± SEM;* n* = 7; *P < 0.05 Sham versus VNS; ^#^ *P* < 0.05 versus time point 0 min. *Bottom:* Area under the curve (AUC) of the glucose tolerance tests (GTT) during sham stimulation (Sham) or vagal nerve stimulation (VNS). In all seven animals the AUC of the GTT was larger during VNS than during sham stimulation. Numbers next to the circles for VNS are animal numbers according to Table 1 and figure legend. Values are means ± SEM;* n* = 7; *P < 0.05 Sham versus VNS.

Compared to sham stimulation, fasted blood glucose levels before the start of the glucose tolerance tests were significantly elevated by VNS (+31 ± 9 mg/dL, *n* = 7, *P* < 0.05). During sham stimulation, blood glucose levels reached a peak value within 15 min following glucose administration and then returned to levels not significantly different from baseline within 90 min. In contrast, during VNS, peak levels were reached only after 30 min and the blood glucose concentration did not return to baseline levels during the 2‐h period of the glucose tolerance tests. During that time period (30–120 min), blood glucose levels were significantly higher during VNS compared to sham stimulation (Fig. 2, top). Importantly, regardless of the genotype or metabolic and hemodynamic phenotype, in all seven animals, the area under the curve of the blood glucose concentration during the 2‐h glucose tolerance tests was greater during VNS than during sham stimulation (Fig. 2, bottom).

### Effects of VNS on insulin and glucagon

**Figure 3:**
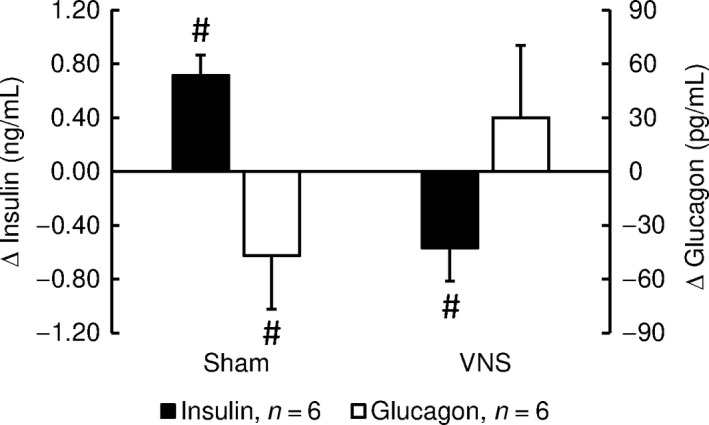
Serum insulin (black bars) and glucagon (white bars) responses to glucose administration. Values are differences between 1‐h and baseline values of glucose tolerance tests. While serum insulin concentration increased in response to glucose administration during sham stimulation, it decreased during VNS. Values are means ± SEM;* n* = 6; ^#^ *P* < 0.05 for absolute 1‐h values versus absolute baseline values (before glucose administration).

Fasted serum insulin and glucagon concentrations (before glucose administration) were not significantly different during VNS and sham stimulation, although there was a trend of higher insulin (*P* = 0.12) concentrations with VNS (Table 4). One hour into the glucose tolerance test, insulin had significantly increased (+0.71 ± 0.15 ng/mL, *P* < 0.05) and glucagon decreased (−47 ± 30 pg/mL, *P* < 0.05) in the sham‐stimulated condition. In contrast, during VNS, insulin significantly decreased (−0.57 ± 0.25 ng/mL, *P* < 0.05) while glucagon did not change significantly (Table 4 and Fig. 3).

**Table 4:** Glucose, insulin, and glucagon responses in glucose tolerance tests

	Sham stimulation			Vagal nerve stimulation		
	Glucose (mg/dL)	Insulin (ng/mL)	Glucagon (pg/mL)	Glucose (mg/dL)	Insulin (ng/mL)	Glucagon (pg/mL)
Baseline	102 ± 5	1.13 ± 0.32	99.5 ± 36.4	134 ± 13*	1.93 ± 0.53	65.0 ± 17.1
1‐h	252 ± 42^#^	1.84 ± 0.42^#^	52.7 ± 21.2^#^	493 ± 36^#^*	1.37 ± 0.38^#^	95.0 ± 47.1

Glucose, insulin, and glucagon serum concentrations before (baseline) and 1 h after glucose administration in the glucose tolerance tests during sham stimulation (left columns) and vagal nerve stimulation (right columns). Values are means ± SEM; *n* = 6; *P < 0.05 VNS versus sham stimulation; ^#^ *P* < 0.05 1‐h value versus baseline value.

## Discussion

The major finding of this study is that cervical VNS of the intact vagus nerve impairs glucose tolerance and inhibits glucose‐induced insulin release in conscious rats. These findings are based on the glucose tolerance tests that consistently resulted in higher peak glucose concentrations and prolonged hyperglycemia following intraperitoneal dextrose application during VNS compared to sham stimulation (Fig. 2). Importantly, this hyperglycemic response was associated with a significant decrease in insulin serum concentration during VNS, instead of the expected increase in insulin serum concentration as observed during sham stimulation (Fig. 3). Thus, the exaggerated hyperglycemia in response to the glucose challenge during VNS may be explained by VNS‐induced inhibition of glucose‐stimulated pancreatic insulin secretion. Our previous study (Meyers et al. 2016) demonstrated that selective afferent cervical VNS evokes a marked and sustained increase in blood glucose concentration in anesthetized rats without concomitant increase in insulin serum concentration. In contrast, selective efferent cervical VNS did not suppress insulin release and, thus increased blood glucose levels only temporarily. Based on these previous findings, it is reasonable to conclude that the inhibition of glucose‐induced insulin release by cervical VNS observed in our current study is mediated through the activation of the afferent rather than efferent nerve fibers within the cervical vagus nerve. Because our previous study demonstrated differential hemodynamic and respiratory responses to right and left cervical VNS (Stauss 2017), we randomly stimulated either the right or left cervical vagus nerve in our current study. However, inhibition of glucose‐induced insulin release was found in rats with left‐ and right‐sided cervical VNS indicating that afferent fibers in both cervical vagal nerves mediate impaired glucose tolerance.

An important aspect of our study is that a mixed population of aged (6–12 months) rats from the Lyon strain (Dupont et al. 1973) was used that ranged from lean and normotensive to obese and hypertensive animals (e.g., rats 1 vs. 7 in Table 1). The Lyon hypertensive rats (LH) differ from the Lyon normotensive (LN) and Lyon hypotensive (LL) rats by development of hypertension, obesity, hyperinsulinemia associated with increased insulin to glucose ratio, increased plasma lipids, and proteinuria with elevated creatinine and urea levels (Sassolas et al. 1981; Vincent et al. 1996). Thus, LH rats express risk factors consistent with metabolic syndrome including insulin resistance. Consistent with insulin resistance in LH rats we found 45% higher fasted insulin serum concentrations in congenic LH rats (1.26 ± 0.44 ng/mL, *n* = 4) compared to LL rats (0.87 ± 0.54 ng/mL, *n* = 2), while fasted blood glucose levels were similar (103 ± 7 mg/dL; *n* = 4 in LH vs. 100 ± 2 mg/dL; *n* = 2 in LL). Based on the insulin responses to intravenous glucose tolerance tests, Boulanger et al. (1997) concluded that LH rats do not develop true insulin resistance at 22 and 52 weeks of age compared to LN rats. However, consistent with our data, these investigators also reported elevated baseline plasma insulin levels and similar baseline glucose levels in LH versus LN rats (Boulanger et al. 1997). Contrary to the initial breeding strategy (Dupont et al. 1973), Lyon rats from the hypotensive (LL) strain were not hypotensive in the current study (Table 1), which is consistent with other reports on the LL strain (Liu et al. 2006; Li et al. 2007). The rationale for using a mixed population of rats was that it allowed us to test if the effects of cervical VNS on glucose metabolism are consistent across a variety of rat strains with differing hemodynamic and metabolic phenotypes. However, it is also important to note that the study was not designed to investigate the specific effects of these different phenotypes on the responses to VNS. Nevertheless, despite the highly heterogeneous hemodynamic and metabolic phenotypes of the rats included in this study we obtained highly consistent responses to VNS. In each individual animal (without exception), the area under the curve of the glucose tolerance tests was greater during VNS than during sham VNS. Likewise, the insulin response was highly consistent. In all but one rat (rat 4 in Table 1), the insulin serum concentration decreased following glucose administration during VNS, while the insulin levels consistently increased in all rats during sham stimulation. In the one rat in which insulin did not decrease with VNS insulin still increased less (+0.24 ng/mL) during VNS than during sham stimulation (+0.49 ng/mL). Thus, the inhibition of glucose‐induced insulin release by cervical VNS was highly consistent in our mixed population of rats suggesting that VNS impairs glucose tolerance in subjects with (e.g., congenic LH rats) and without (e.g., LN or LL rats) hyperinsulinemia or other metabolic risk factors. This observation raises the question if patients without previous metabolic risk factors may develop glucose intolerance following initiation of cervical VNS treatment or if VNS may drive patients with prediabetes into a state of overt diabetes.

While our data demonstrate that cervical VNS can cause hyperglycemia through inhibition of glucose‐induced insulin release, mechanisms other than inhibition of insulin or stimulation of glucagon release likely contributed to the elevated fasted blood glucose levels with VNS. That is because in the fasted state (before glucose administration) neither serum insulin levels were reduced nor glucagon levels were increased compared to sham stimulation (Table 4). To explore the possibility that cervical VNS increases fasted blood glucose levels through an activation of the sympathetic nervous system, we performed heart rate variability analysis. This analysis revealed that cervical VNS was associated with elevated low‐ (LF) and high‐frequency (HF) heart rate variability (Table 3) with no change in the LF/HF ratio (data not shown). It is generally accepted that LF heart rate variability reflects sympathetic and to some extent also parasympathetic modulation of cardiac function, while HF heart rate variability is exclusively driven by the parasympathetic nervous system (Task Force of the European Society of Cardiology and the North American Society of Pacing and Electrophysiology, 1996; Stauss 2007). Since the LF/HF ratio was not altered and the absolute spectral power of both LF and HF heart rate variability increased significantly by VNS, one may conclude that cervical VNS causes parallel increases in cardiac sympathetic and parasympathetic modulation. Thus, one may speculate that cervical VNS may also increase the sympathetic modulation of metabolic function and potentially increase fasted blood glucose levels through a sympathetic‐mediated increase in hepatic glucose release. However, further studies utilizing more direct measures of sympathetic tone or possibly sympathetic blockade experiments need to be conducted to verify such a potential mechanism.

While the results of this study are potentially relevant regarding the possibility of an increased risk for glucose intolerance and associated metabolic derangements in patients treated with cervical VNS, to the best of our knowledge, no clinical study directly investigated this possibility so far. In nondiabetic patients treated with vagal nerve blockade for obesity, no acute effects of vagal nerve blockade were observed on glucose metabolism and insulin secretion (Sathananthan et al. 2014). However, this finding does not mean that cervical VNS does not affect glucose metabolism in humans, because in these patients, the subdiaphragmatic vagal nerve fibers were blocked that may not contain the same fibers responsible for the metabolic responses seen with cervical VNS in our animal model. Furthermore, blocking the vagal nerve traffic using kilohertz stimulation may not activate afferent nerve fibers that are likely to mediate the hyperglycemic response observed in our study. There is also some limited evidence that noninvasive transcutaneous auricular vagus nerve stimulation (taVNS) may improve glucose metabolism in humans (Huang et al. 2014). In this study lower fasted and 2 h postprandial blood glucose levels and reduced HbA1c levels were observed following 12 weeks of taVNS (20 min twice a day) compared to a group of subjects without treatment. However, a sham‐stimulated control group had similar 2 h postprandial glucose responses than the taVNS group. Of course, stimulating the auricular branch of the vagus nerve through taVNS activates different nerve fibers than cervical VNS. Therefore, taVNS and cervical VNS may affect glucose metabolism differently. The translatability of our data on the effects of cervical VNS on glucose metabolism in rats to the situation in patients treated with cervical VNS is limited by several factors. First, vagal nerve fibers within the cervical vagus nerve may differ in rats and humans, and the specific fibers mediating the response in rats may not travel within the cervical vagus nerve in humans. Second, the stimulation parameters used in our study are different from the stimulation parameters used in patients. Particularly we used a duty cycle of 1 h of stimulation followed by 1 h without stimulation which allowed us to verify successful VNS by the periodic fluctuations in heart rate and blood pressure shown in Figure 1. In most patients the stimulators are programmed for cycles of 30 sec of stimulation followed by 5 min without stimulation. Thus during a 24 h period the rats would have been stimulated for 12 h, whereas human patients would have been stimulated for only 2 h and 11 min. The shorter cumulative stimulation period in humans compared to the rats in this study may limit the adverse effects of cervical VNS on metabolic function in patients. Furthermore, 30 sec of stimulation may not be long enough to effectively inhibit insulin and the “off cycle” of 5 min without stimulation may be enough to raise insulin in response to a glucose challenge.

In conclusion, this study demonstrates that cervical VNS impairs glucose tolerance and raises fasted blood glucose levels in conscious rats. These effects occur in response to stimulation of either the right or left cervical vagus nerve and are likely mediated by afferent signaling within the vagus nerve. Sympathetic‐mediated hepatic glucose release may contribute to the increased fasted blood glucose levels in response to cervical VNS. Unless clinical studies are conducted that refute a potential adverse effect of cervical VNS on glucose metabolism in patients treated with VNS, glucose metabolism should be monitored regularly in such patients.

## Conflict of Interest

Harald Stauss was the founder of Harald Stauss Scientific, a company that marketed implantable vagal nerve stimulators for rodents. The company is no longer active and the stimulators may now be obtained through a Material Transfer Agreement with Harald Stauss at the Burrell College of Osteopathic Medicine.
